# Advancing Accuracy in Multimodal Medical Tasks Through Bootstrapped Language-Image Pretraining (BioMedBLIP): Performance Evaluation Study

**DOI:** 10.2196/56627

**Published:** 2024-08-05

**Authors:** Usman Naseem, Surendrabikram Thapa, Anum Masood

**Affiliations:** 1 School of Computing Macquarie University Sydney Australia; 2 Department of Computer Science Virginia Tech Blacksburg, VA United States; 3 Department of Circulation and Medical Imaging Norwegian University of Science and Technology Trondheim Norway; 4 Harvard Medical School Harvard University Boston, MA United States; 5 Department of Radiology Boston Children's Hospital Boston, MA United States

**Keywords:** biomedical text mining, BioNLP, vision-language pretraining, multimodal models, medical image analysis

## Abstract

**Background:**

Medical image analysis, particularly in the context of visual question answering (VQA) and image captioning, is crucial for accurate diagnosis and educational purposes.

**Objective:**

Our study aims to introduce BioMedBLIP models, fine-tuned for VQA tasks using specialized medical data sets such as Radiology Objects in Context and Medical Information Mart for Intensive Care-Chest X-ray, and evaluate their performance in comparison to the state of the art (SOTA) original Bootstrapping Language-Image Pretraining (BLIP) model.

**Methods:**

We present 9 versions of BioMedBLIP across 3 downstream tasks in various data sets. The models are trained on a varying number of epochs. The findings indicate the strong overall performance of our models. We proposed BioMedBLIP for the VQA generation model, VQA classification model, and BioMedBLIP image caption model. We conducted pretraining in BLIP using medical data sets, producing an adapted BLIP model tailored for medical applications.

**Results:**

In VQA generation tasks, BioMedBLIP models outperformed the SOTA on the Semantically-Labeled Knowledge-Enhanced (SLAKE) data set, VQA in Radiology (VQA-RAD), and Image Cross-Language Evaluation Forum data sets. In VQA classification, our models consistently surpassed the SOTA on the SLAKE data set. Our models also showed competitive performance on the VQA-RAD and PathVQA data sets. Similarly, in image captioning tasks, our model beat the SOTA, suggesting the importance of pretraining with medical data sets. Overall, in 20 different data sets and task combinations, our BioMedBLIP excelled in 15 (75%) out of 20 tasks. BioMedBLIP represents a new SOTA in 15 (75%) out of 20 tasks, and our responses were rated higher in all 20 tasks (*P*<.005) in comparison to SOTA models.

**Conclusions:**

Our BioMedBLIP models show promising performance and suggest that incorporating medical knowledge through pretraining with domain-specific medical data sets helps models achieve higher performance. Our models thus demonstrate their potential to advance medical image analysis, impacting diagnosis, medical education, and research. However, data quality, task-specific variability, computational resources, and ethical considerations should be carefully addressed. In conclusion, our models represent a contribution toward the synergy of artificial intelligence and medicine. We have made BioMedBLIP freely available, which will help in further advancing research in multimodal medical tasks.

## Introduction

### Background

In recent decades, the fields of data analysis, machine learning, and deep learning have undergone remarkable advancements, with profound implications for various professional domains [1,2]. One of the most promising frontiers for these advancements is medical science, where data-driven models have the potential to bring about significant breakthroughs [3,4]. Medical data predominantly exist in the form of images and textual reports, encompassing x-ray images, medical records, and more. To harness the full potential of these data sources, a visual language model capable of extracting insights from both images and text becomes paramount. Visual language models, which are at the core of this research, represent a fusion of computer vision and natural language processing (NLP). These models possess the capability to understand and generate text-based descriptions for visual content, making them invaluable in contexts where both images and text are essential for comprehensive analysis.

This study explored and adapted visual language models specifically for medical data sets, building upon the foundation laid by existing models. The primary objective was to enhance the performance of these models when confronted with medical data, such as the Radiology Objects in Context (ROCO) [5] and Medical Information Mart for Intensive Care-Chest X-ray (MIMIC-CXR) [6] data sets. This was achieved through a comprehensive process of pretraining on medical data sets and rigorous fine-tuning, with the ultimate goal of determining the optimal model configurations and parameters. This study thus facilitates advancements in health care, contributing to more accurate diagnoses; streamlined medical reporting; and, ultimately, improved patient care. We have made BioMedBLIP models freely available, facilitating the progress of research in diverse medical applications involving multiple modalities [7].

### Related Work

In the domain of visual language models and their applications within the medical field, several notable studies and advancements have paved the way for this research project. These works solve different problems within health care analytics and have played critical roles in shaping this study’s foundation.

Within the medical domain, image captioning has emerged as a valuable tool that enables health care professionals and researchers to enhance their diagnostic and reporting processes. Image captioning technology allows for the automatic generation of textual descriptions for medical images, such as x-rays, magnetic resonance imaging (MRI) scans, and computed tomography (CT) scans. This capability brings about several significant benefits. First, it aids clinicians in the diagnostic process by providing detailed descriptions of medical images, helping medical professionals to quickly and accurately identify abnormalities or pathologies in the images, thus improving the efficiency and accuracy of diagnoses. Second, image captions serve as a means of clear and standardized communication among health care professionals, reducing the potential for misinterpretation when multiple experts are involved in the diagnostic process. Third, image captions make medical images more accessible to a broader audience, including patients, promoting health literacy and patient engagement. Moreover, in a clinical setting, image captions expedite the process of creating medical reports, improving the overall quality of patient records. They also play a valuable role in medical education and training, aiding in the learning and teaching of medical imaging and diagnostics. Pavlopoulos et al [8] proposed that biomedical image captioning can significantly expedite clinicians’ diagnostic processes and presented a comprehensive survey covering various aspects of medical image captioning, including data sets and evaluation measures. Furthermore, the task of the automatic generation of medical image reports, introduced by Jing et al [9], aimed to streamline the reporting process for physicians, enhancing both efficiency and accuracy. To address this, Jing et al [9] used a hierarchical Long Short-Term Memory model, which was tested on 2 publicly available data sets, Indiana University X-Ray [10] and Pathology Education Informational Resource (PEIR)-Gross [9].

This connection between image captioning and medical report generation underscores the practical utility of visual language models in improving health care processes. In addition to these advancements, the field of medical visual question answering (VQA) has gained increasing relevance. Medical VQA tasks involve developing models capable of answering questions related to medical images and bridging the gap between textual queries and visual data. Lin et al [11] introduced various medical data sets and proposed methods to enhance model performance in medical VQA tasks. We used various data sets presented by Lin et al [11] in our experiments. Furthermore, Li et al [12] emphasized the significance of pretraining models on general images to capture meaningful representations of medical data, thus laying the groundwork for our approach. This insight served as our motivation to explore an approach of pretraining models on domain-specific medical data sets, with the aim of achieving enhanced performance for medical VQA (MedVQA) tasks. Notably, Li et al [12] encountered a limitation in their work, as they did not pretrain models using medical data sets. Their decision was influenced by computational resource constraints, and they believed that domain-specific pretraining would be the key to improving model performance in MedVQA tasks. To address this gap in the research landscape, we took the initiative to pretrain our model using medical data sets, thereby bridging the gap between general and medical image understanding.

Transformer models have become instrumental in a diverse array of applications in various vision and language (V+L) tasks, including medical VQA. The Transformer, proposed by Vaswani et al [13], represents a departure from traditional recurrent or convolutional neural networks. Its architecture replaces recurrent layers with a multihead self-attention encoder and decoder structure. Compared to traditional recurrent neural network models, the Transformer significantly reduces training time, making it a scalable solution capable of handling a wide range of inputs and applicable to diverse V+L tasks, including the analysis of medical images.

Several prominent transformer-based models have had a significant impact on the landscape of NLP and multimodal tasks. One of the most influential models is Bidirectional Encoder Representations From Transformers (BERT). Proposed by Devlin et al [14], BERT has demonstrated its efficacy in a wide variety of NLP tasks. This is achieved through a pretraining phase where 15% of input sequences are masked. These masked tokens can be replaced with random words, original words, or MASK tokens. Subsequently, the transformer auto-encodes these tokens, and fine-tuning is applied to adapt the pretrained model to downstream tasks. The generalization capabilities of BERT are remarkable, making it adept at handling a wide array of semantic tasks. This is due in part to its bidirectional training, which allows the model to learn contextual information from both the left and right sides of a given word.

BERT’s versatility allows it to be tailored for various applications, and one domain where it has shown great promise is the biomedical field. In the biomedical domain, text data often exhibit complex language patterns and domain-specific terminology. Lee et al [15] recognized the need for a model that could adapt to these linguistic intricacies and introduced BioBERT, a BERT-based model fine-tuned on biomedical text. BioBERT effectively addresses the word distribution shift from general data to biomedical data, making it a valuable tool for tasks such as biomedical text mining. The BioBERT model’s workflow involves transferring weights from BERT, which is pretrained on general domain data, to BioBERT. Subsequently, BioBERT is pretrained on biomedical domain data, followed by fine-tuning and evaluation of various downstream tasks. This adaptation enhances BioBERT’s performance in domain-specific tasks, such as biomedical text classification and named entity recognition.

The success of BERT and its adaptations has paved the way for exploring their application in multimodal tasks, where both text and image data are involved. For instance, VisualBERT, proposed by Li et al [16], was inspired by BERT and designed to capture rich semantics in V+L tasks. It uses a stack of Transformer layers and integrates pretrained object proposal systems for image feature extraction. In the training process, VisualBERT uses self-supervised learning with masked word tokens and performs image caption classification tasks with true and false captions. This approach enables the model to capture intricate relationships between text and image content, making it highly suitable for multimodal tasks where textual descriptions are needed for visual content.

Learning Cross-Modality Encoder Representations from Transformers (LXMERT), another notable model, builds upon the success of BERT and its variants [17]. Tan and Bansal [17] recognized the importance of interpreting the semantic meaning of both images and text while exploring the relationships between V+L. LXMERT’s encoders, based on the Transformer architecture, are pretrained on large volumes of image-text pairs. The pretraining process, inspired by BERT, includes techniques such as adding random masks. Interestingly, LXMERT’s pretraining approach has been found to outperform data augmentation, a common practice used to increase the amount of training data. Consequently, LXMERT is well suited for tasks that involve understanding and generating textual descriptions for visual content, such as image captioning and VQA.

As the field of V+L tasks continues to evolve, so do the transformer-based models designed to tackle them. The Vision Transformer (ViT), introduced by Dosovitskiy et al [18], represents an innovation to address the challenges of applying the Transformer architecture directly to computer vision tasks. ViT operates by dividing an image into 16×16 patches and processing them with position embeddings using a standard Transformer encoder. This approach has shown promise, but it demands substantial computational resources and extensive training data. Notably, ViT32 ViT was used by Eslami et al [19] as part of fine-tuned versions of Contrastive Language-Image Pretraining (CLIP), comparing the performance of different models in the medical domain.

Similarly, Universal Image-Text Representation (UNITER), proposed by Chen et al [20], takes inspiration from the BERT model. The UNITER model has demonstrated strong performance in various V+L tasks. The architecture of UNITER uses the Transformer as its core, with the image and text embedder working in tandem to encode image and text features into a common embedding space. This approach enables the generation of contextual embeddings, facilitating a better understanding of the relationships of V+L. These transformer-based models collectively represent a spectrum of approaches and adaptations within the broader field of V+L tasks.

The landscape of transformer-based models, ranging from BERT to ViT, demonstrates their adaptability and effectiveness in various domains, including NLP and multimodal tasks. CLIP, introduced by Radford et al [21], represents a significant step forward in the multimodal domain. CLIP was designed to connect images and text through a shared embedding space, enabling it to understand the relationship between the 2 modalities. By pretraining on a massive data set containing images and their associated textual descriptions, CLIP can align images with natural language descriptions, making it a versatile tool for a wide range of tasks. This novel approach has significant implications for the medical field, where visual data, such as medical images, often require textual descriptions for comprehensive analysis and interpretation.

Building upon the success of CLIP, PubMedCLIP emerged as a tailored solution for MedVQA. Eslami et al [19] recognized the need for a model specifically adapted to the medical domain and developed PubMedCLIP, a fine-tuned version of CLIP trained on a data set of medical image-text pairs from PubMed articles. This adaptation enables PubMedCLIP to better understand the nuances of medical images and text, resulting in improved performance on MedVQA tasks.

One of the recent works fusing medical imaging and text data is MedBLIP [22]. MedBLIP uses a trainable 3D vision encoder, MedQFormer, which connects medical images with language models. However, MedBLIP could not significantly improve VQA performance and classification accuracy. For the experimental evaluation of MedBLIP, authors only used MRI scans and text, which will limit the use of MedBLIP in the case of other modalities, such as positron emission tomography, CT, and x-ray images.

Despite performing well in vision-language tasks, CLIP suffers from a number of limitations. Firstly, it is primarily focused on vision-language understanding tasks, such as image retrieval and VQA. This means that it is not well suited for generation tasks, such as image captioning. Secondly, CLIP is trained on a large data set of image-text pairs collected from the web. However, these data are often noisy and contain incorrect or misleading captions. This can lead to CLIP making mistakes when performing tasks that require an accurate understanding of the relationship between images and text.

**Figure 1:**
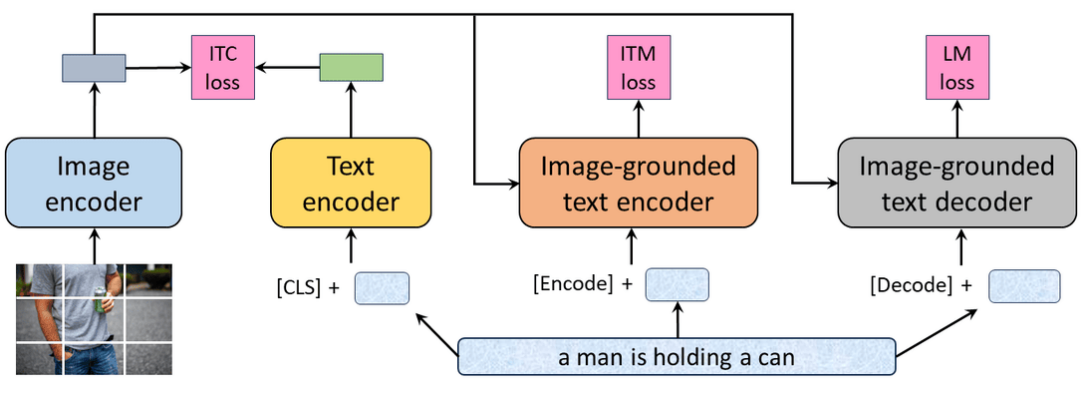
Pretraining architecture of Bootstrapping Language-Image Pretraining (BLIP). CLS: classification; ITC: image-text contrastive; ITM: image-text matching; LM: language modeling.

To address these limitations, Li et al [23] proposed Bootstrapping Language-Image Pretraining (BLIP), a vision-language pretraining framework. BLIP, as shown in Figure 1, is a unified model that can be used for both understanding and generation tasks. This is achieved by incorporating a captioning module into the model, which allows BLIP to generate captions for images. In addition, BLIP addresses the issue of noisy web data by bootstrapping the captions. This means that a captioner generates synthetic captions and a filter removes the noisy ones. This results in a cleaner data set that can be used to train a more robust model. As a result of these improvements, BLIP has been shown to achieve state-of-the-art (SOTA) results on a wide range of vision-language tasks, including image retrieval, VQA, image captioning, and visual grounding. In addition, BLIP is more efficient to train and can be fine-tuned for downstream tasks with lesser data. Finally, BLIP is more interpretable than CLIP, as the captioning module allows users to understand how the model is reasoning about images.

Capitalizing on the strengths of BLIP, we propose BioMedBLIP by pretraining and fine-tuning the model with a medical data set to achieve SOTA results on medical vision-language tasks. Specifically, BLIP’s unified approach to vision-language understanding and generation makes it well suited for tasks such as medical image classification, medical image retrieval, and medical image captioning. In addition, BLIP’s ability to handle noisy data makes it well suited for training on medical data sets, which can often be noisy and contain incomplete or inaccurate information. We evaluated our pretrained model using various standard task-specific performance metrics.

## Methods

In this section, we describe our pretraining process along with training strategies and resources used.

### BioMedBLIP

#### Overview

BLIP, initially pretrained on general image data sets, possesses knowledge rooted in general image understanding. However, medical images exhibit distinct characteristics that differentiate them from general images. Many medical images are gray scale, such as x-rays and MRI scans, which results in a significant divergence between the general image domain and the medical image domain. To bridge this gap, we conducted the pretraining of BLIP using medical data sets, producing an adapted BLIP model tailored for medical applications.

As shown in Figure 1, BLIP is organized into 4 key modules.

- Visual transformer block (image encoder): the first module serves as an image encoder, using a visual transformer to extract features from medical images.
- BERT-based text encoder: the second module is a text encoder based on BERT. It processes textual data, ensuring a comprehensive understanding of medical texts.
- Cross-attention and binary classification: the third module shares parameters with the text encoder, facilitating joint image-text embeddings through cross-attention. It uses a binary classifier to confirm the pairing of images and text.
- Text decoder: the final module is a text decoder, which shares some components with the preceding encoders, such as feed-forward and cross-attention layers. However, it maintains its own causal self-attention layers. The text decoder generates text auto-regressively, and cross-entropy loss is applied during this process.

For BLIP, we explored various pretraining approaches. Initially, we attempted to pretrain BLIP from scratch. Subsequently, we pretrained BLIP from a provided checkpoint using the ROCO and MIMIC data sets. Further experimentation involved extending the checkpoint with the inclusion of the ROCO data set onto the MIMIC data set. To apply BLIP to downstream tasks, we followed the BLIP framework’s process to refactor the model’s modules and assembled an adapted model tailored for specific tasks.

#### BioMedBLIP VQA Generation Model

**Figure 2:**
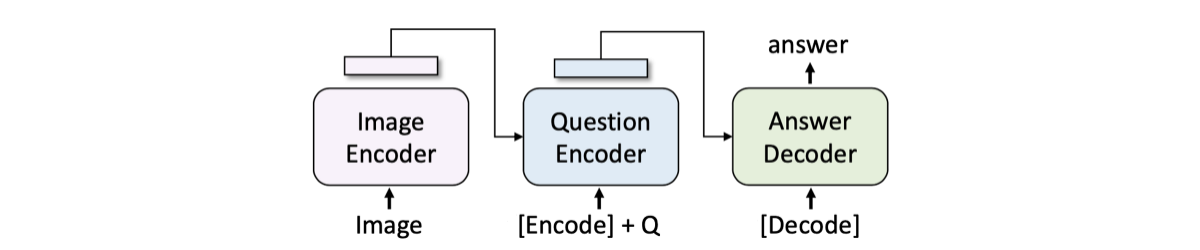
BioMedBLIP visual question answering generation model.

For VQA tasks, we adopted the structure provided by BLIP, as depicted in Figure 2. VQA tasks require the model to generate textual answers based on given images and question pairs. The process involves encoding the image to create image embeddings, producing image-question joint embeddings with the help of the question encoder, and using the answer decoder to generate the final answer.

#### Modified BLIP Classification Model

**Figure 3:**
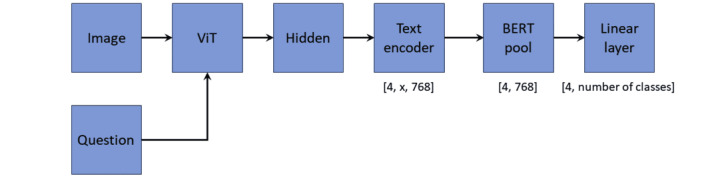
BioMedBLIP classification model. ViT: Vision Transformer.

The modified BLIP classification model, illustrated in Figure 3, shares similarities with the generation model. It generates joint image-text embeddings using the image encoder and text encoder. However, instead of using the answer decoder, a pooling layer is introduced to reduce the vector dimension. Subsequently, a linear classification layer is applied to produce multiple classification results.

#### BioMedBLIP Image Caption Model

**Figure 4:**
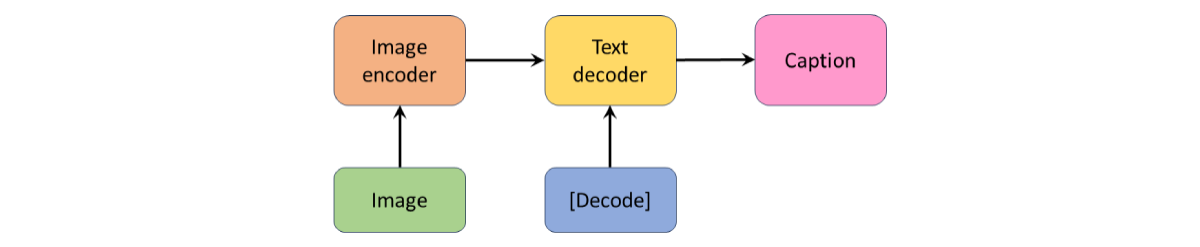
BioMedBLIP image caption model.

The image caption model, presented in Figure 4, is composed of the image encoder and text decoder, following BLIP’s implementation. Unlike VQA tasks, the image captioning task involves generating text based solely on images. Therefore, the text encoder is omitted, and the text decoder takes image embeddings provided by the image encoder and the *[Decode]* token as input to produce image captions.

### Data Sets

Our research leverages a diverse range of medical data sets, encompassing a variety of visual and textual medical data sources. These data sets serve as the foundation for pretraining and fine-tuning our visual language model.

#### ROCO Data Set

**Figure 5:**
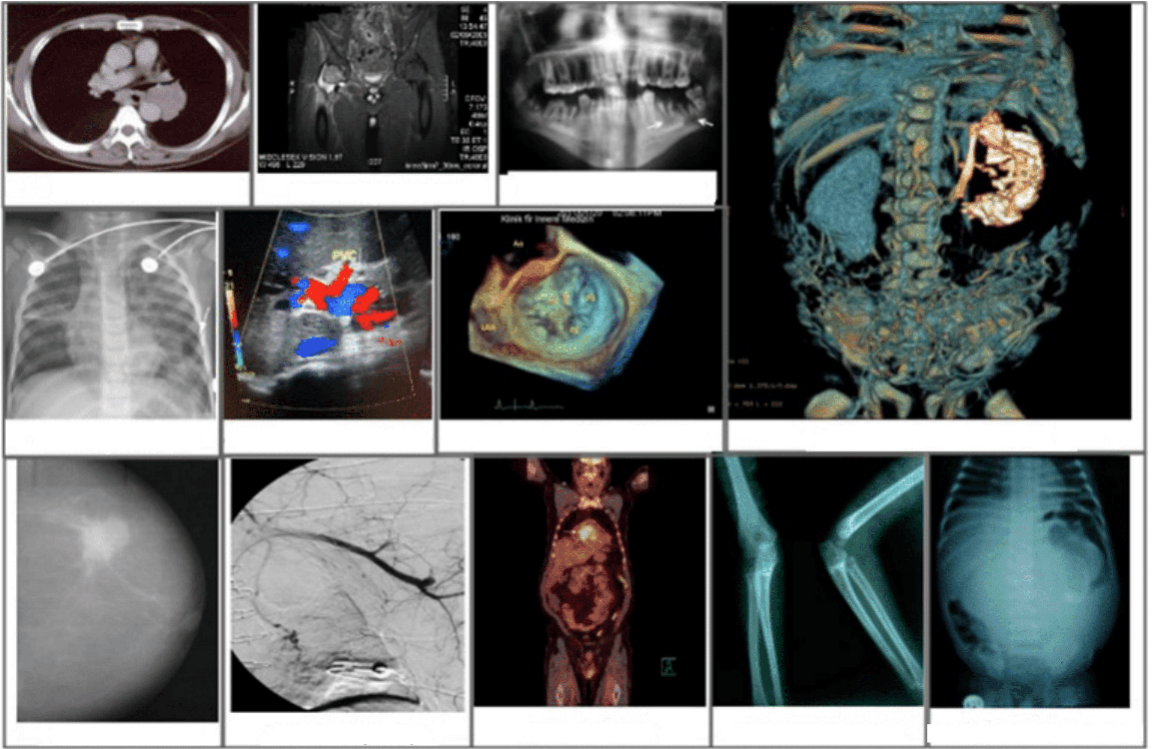
Some images in the Radiology Objects in Context data set.

The ROCO data set [5] plays a pivotal role in pretraining BioMedBLIP. It encompasses over 81,000 radiology images representing multiple medical imaging modalities, including CT, ultrasound, x-ray, fluoroscopy, positron emission tomography, mammography, MRI, and angiography [5]. Our approach involved consolidating the training, validation, and test data into a single comprehensive JSON file, enabling the pretraining of BioMedBLIP. Notably, the captions in the ROCO data set were sourced from peer-reviewed scientific biomedical literature and downloaded from a GitHub link [24]. Some examples are shown in Figure 5.

#### MIMIC-CXR Data Set

**Figure 6:**
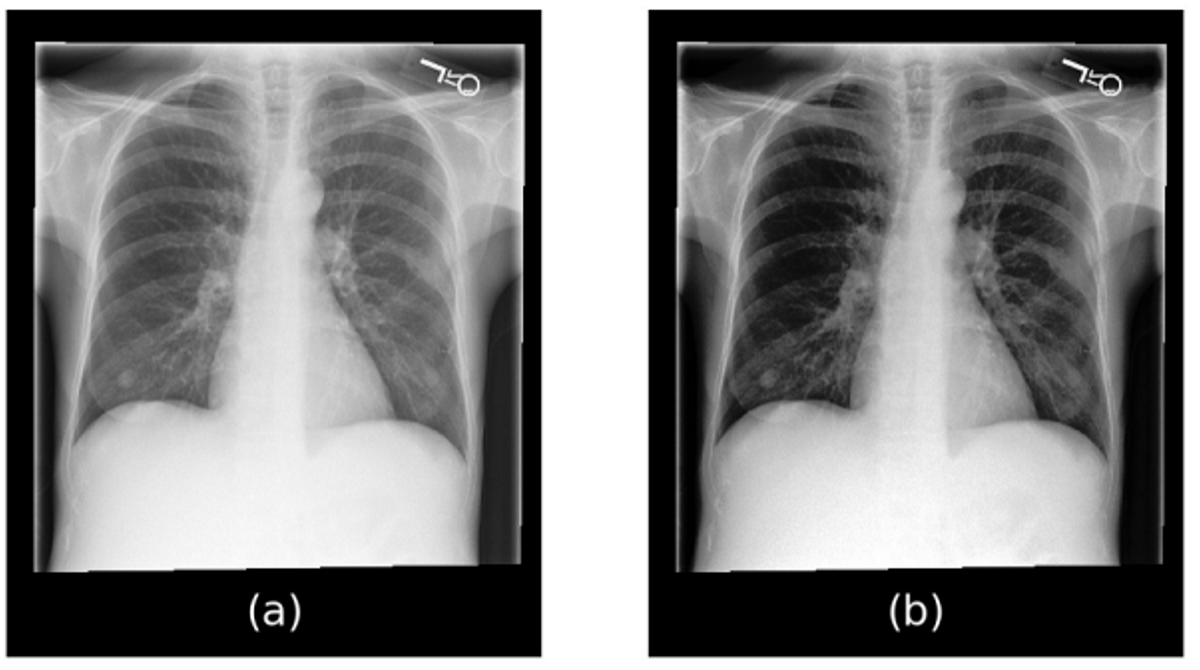
Chest x-rays in the Medical Information Mart for Intensive Care-Chest X-ray data set.

The MIMIC-CXR data set is a large data set that consists of 377,110 chest x-rays corresponding to 227,827 imaging studies [6]. Some examples of chest x-rays from this data set are shown in Figure 6. In our context, we used this data set for BioMedBLIP’s pretraining. It is worth noting that each medical study extracted from the hospital’s electronic health record system can be related to multiple chest x-rays. Our efforts focused on filtering the chest x-rays, retaining those with anteroposterior and posteroanterior positions, and ensuring that each medical report had a single associated chest x-ray. After preprocessing, we obtained 218,139 image-caption pairs, which were instrumental in pretraining BioMedBLIP. The medical studies are XML files, and we extracted the “Findings” and “Impressions” sections as the caption for medical images.

#### Image Cross-Language Evaluation Forum 2019 Data Set

**Figure 7:**
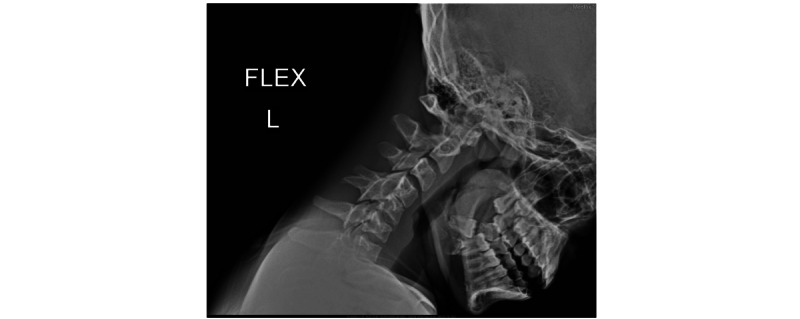
A radiology image from the Image Cross-Language Evaluation Forum 2019 data set.

The Image Cross-Language Evaluation Forum (ImageCLEF) 2019 data set [25,26], provided by the ImageCLEF organization for evaluation, served as a critical component in our work. This data set comes in 3 parts: training, validation, and testing sets. No preprocessing was performed on the data set, and it was leveraged for our VQA generation task. It contains 12,792; 2000; and 500 image-caption pairs for the training, validation, and testing sets, respectively. An example of a radiology image in the ImageCLEF data set is shown in Figure 7.

#### Semantically-Labeled Knowledge-Enhanced Data Set

**Figure 8:**
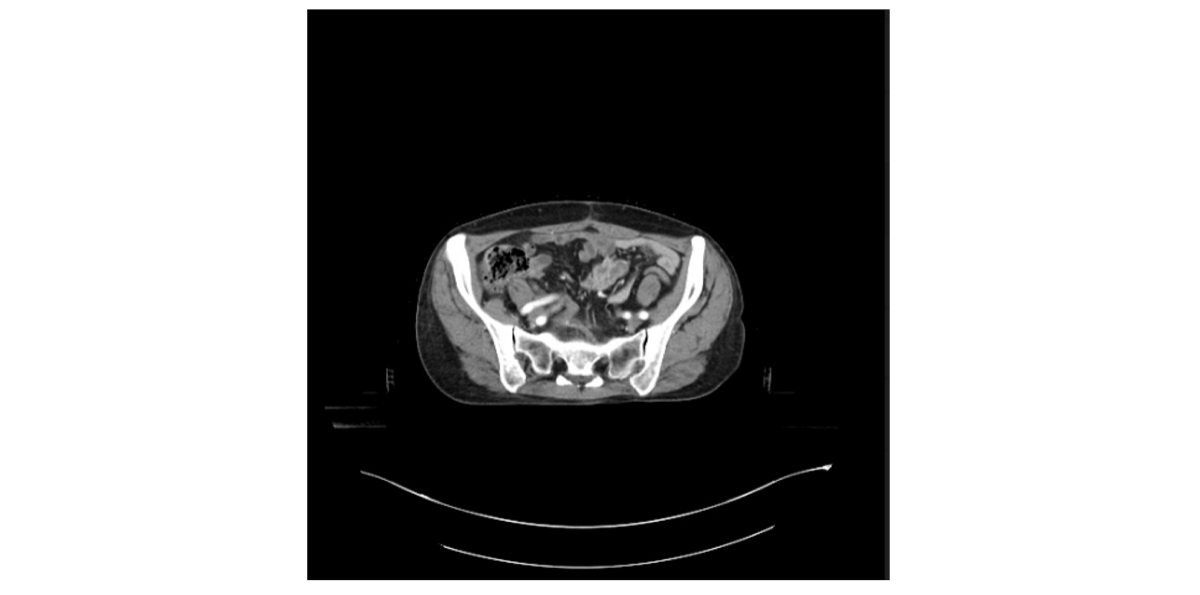
A radiology image from the Semantically-Labeled Knowledge-Enhanced data set.

The Semantically-Labeled Knowledge-Enhanced (SLAKE) data set [27] was designed for medical VQA tasks [28]. We used this data set for VQA generation and VQA classification tasks. The SLAKE data set has both Chinese question-answer pairs and English question-answer pairs. Our data set preparation included filtering to retain only English question-answer pairs. After filtering, the SLAKE data set consisted of 4919, 1053, and 1061 image-caption pairs for the training, validation, and testing sets, respectively. Notably, the SLAKE data set features 2 different answer types, open and close, allowing us to assess model performance for open-ended and close-ended questions. An example of a radiology image from the SLAKE data set is shown in Figure 8.

#### PathVQA Data Set

**Figure 9:**
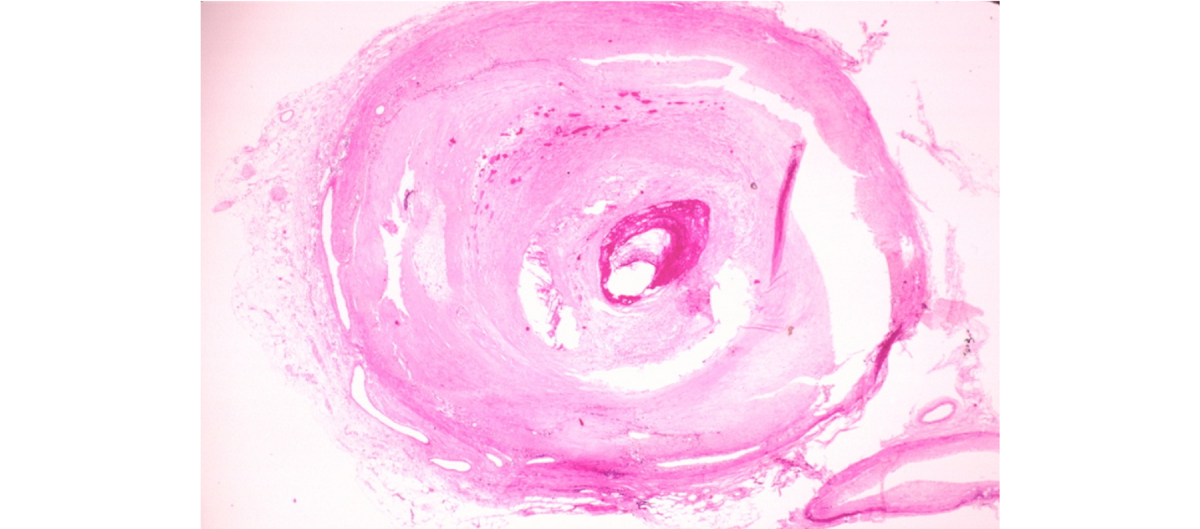
A pathology image from the PathVQA data set.

The PathVQA data set is a VQA data set for “AI pathologist” development [29]. It contains numerous pathology images together with questions and corresponding answers. All image-question-answer triplets are manually checked to ensure correctness. In our setting, we obtained a data set with 32,795 image-question-answer pairs after initial preprocessing. We further categorized questions into open-ended and close-ended questions using our own splitting, yielding 20,968; 5241; and 6552 image-question-answer pairs for the training, validation, and testing sets, respectively. All these images in the validation and testing sets were picked randomly. An example of a pathology image from the PathVQA data set is shown in Figure 9. We used this data set to perform the VQA generation and VQA classification tasks in our project.

#### VQA-RAD Data Set

**Figure 10:**
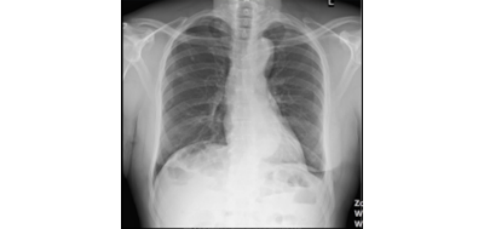
A radiology image from the Visual Question Answering in Radiology data set.

VQA-RAD is the first data set that was manually constructed. During the data collection process, clinicians asked natural questions about radiology images. Meanwhile, their reference answers would be provided [30]. It has radiology images together with question-answer pairs. We used the original data [31] splitting and did not perform any data preprocessing on this data set. It contains 2452, 614, and 452 image-question-answer pairs for the training, validation, and testing sets, respectively. An example of a radiology image from the VQA-RAD data set is shown in Figure 10. We used the VQA-RAD data set to implement the VQA generation and VQA classification tasks.

#### Open-I Data Set

**Figure 11:**
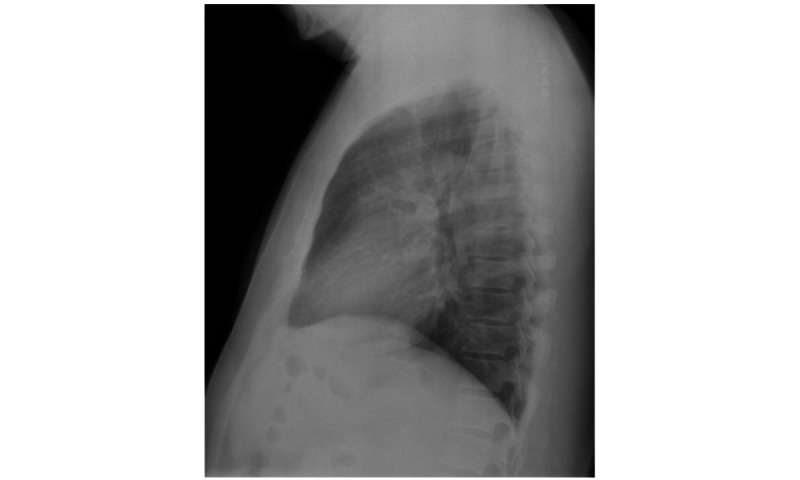
A radiology image from the Open-I data set.

The Open-I data set [32] is a compilation of chest x-ray images collected from open-source literature and biomedical image collections. Our focus was specifically on the chest x-ray images within this data set. We downloaded the data set from the official Open-I website, which comprises 2 parts: images and medical reports. The medical reports were stored as XML files, with the “Finding” and “Impression” sections extracted as captions for the images. Our downloaded version contained 2452, 614, and 452 image-caption pairs for the training, validation, and testing sets, respectively. An example of a radiology image from the Open-I data set is shown in Figure 11.

#### PEIR-Gross Data Set

**Figure 12:**
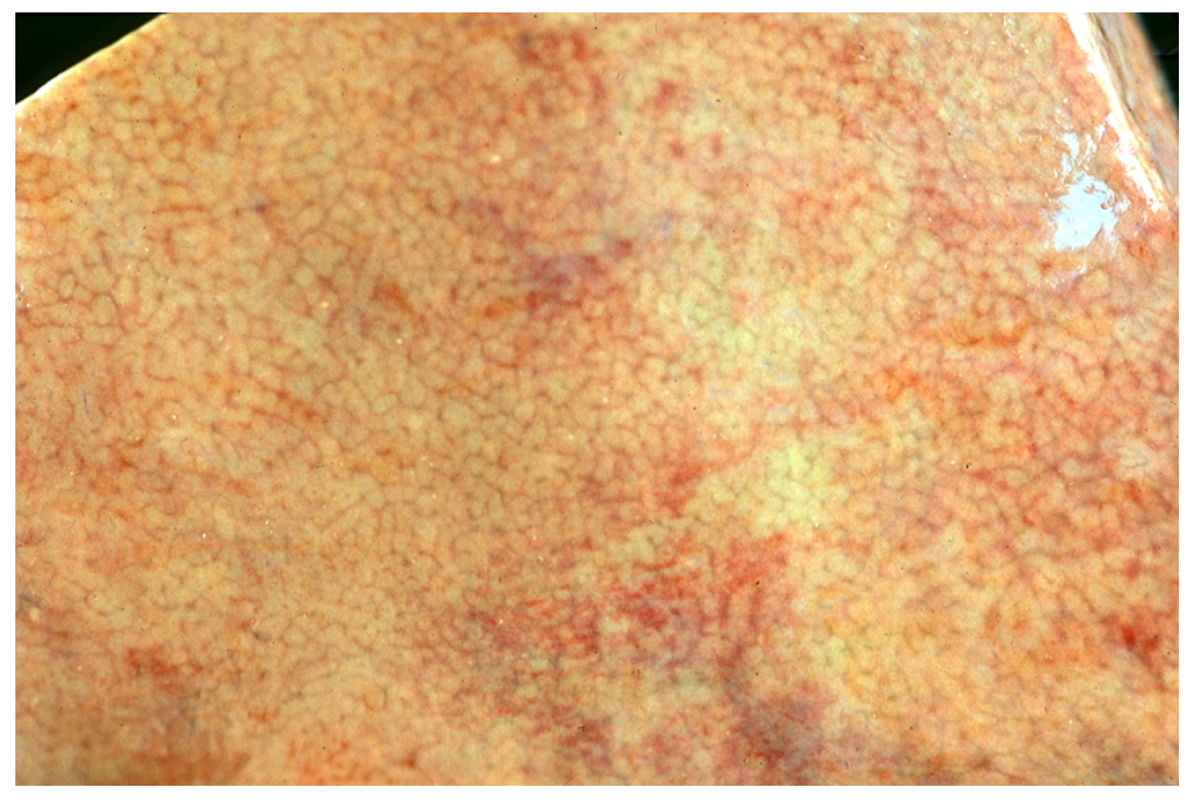
A medical image from Pathology Education Informational Resource-Gross data set.

The PEIR-Gross data set originated from the PEIR and contains 7442 image-caption pairs across 21 subcategories [9]. Our data set preparation involved splitting it into training and testing sets. We also generated a validation set by randomly selecting 10% of the training data. After preprocessing, we had 6029, 669, and 745 image-caption pairs for the training, validation, and testing sets, respectively. An example of a medical image from the PEIR-Gross data set is shown in Figure 12. This data set was used for image captioning tasks.

### Implementation

#### Overview

In addition to the original BLIP base model checkpoint obtained from the original BLIP GitHub repository [33], we also meticulously pretrained a series of checkpoints on various medical data sets. These checkpoints serve as critical resources that we are making available for the broader research community and our clients. Here, we provide a comprehensive list of these checkpoints:

- Original BLIP base model checkpoint (ViT-Base): this checkpoint represents the foundational BLIP model pretrained on the LAION115M data set.
- Pretraining on combined-MED checkpoints: these checkpoints were derived from pretraining on a comprehensive data set combining SLAKE, Open-I, ImageCLEF, and PathVQA data sets from scratch, with versions available for both 20 and 50 epochs.
- Pretraining on SLAKE checkpoint: this checkpoint is the result of pretraining on the SLAKE data set from scratch, spanning 20 epochs.
- Pretraining on ROCO checkpoint: pretrained on the ROCO data set from scratch, this checkpoint encapsulates knowledge gained over 10 epochs.
- Pretraining on MIMIC-CXR checkpoint: for up to 10 epochs, this checkpoint embodies the insights obtained from pretraining on the MIMIC-CXR data set from scratch.
- Pretraining on ROCO from the BLIP original checkpoint: this checkpoint extends the pretraining on the ROCO data set from the existing BLIP checkpoint up to 50 epochs.
- Pretraining on MIMIC-CXR from the BLIP original checkpoint: similar to the ROCO extension, this checkpoint involves the pretraining on the MIMIC-CXR data set from the original BLIP checkpoint and spans up to 50 epochs.
- Pretraining on ROCO and MIMIC-CXR from the BLIP original checkpoint: this checkpoint represents an amalgamation of knowledge acquired from both ROCO and MIMIC-CXR data sets, building upon the original BLIP checkpoint and extending to 50 epochs.

#### Pretraining Details

To undertake the pretraining of the BLIP model, our approach involved several key steps. Initially, we used the ROCO data set for the pretraining of the BLIP original checkpoint, resulting in the creation of the BioMedBLIP-ROCO models. These models were uniquely identified by the number of epochs they were pretrained for, with “BioMedBLIP-ROCO-10,” for instance, signifying the original BLIP checkpoint pretrained on the ROCO data set for 10 epochs. This choice was informed by the fact that the original BLIP checkpoint had been trained on millions of standard images and, therefore, already possessed the fundamental knowledge required for a visual language model. Our attempt to pretrain BLIP from scratch yielded unsatisfactory performance. Subsequently, we embarked on pretraining the original BLIP checkpoint with the MIMIC-CXR data set to produce the BLIP-MIMIC models. Finally, we took the BLIP-MIMIC checkpoints and further pretrained them using the ROCO data set, resulting in the creation of the BioMedBLIP-ROCO and MIMIC models. In this study, we retained checkpoints representing 10, 20, and 50 epochs for each of these models.

To optimize the pretraining process, we conducted a series of experiments aimed at identifying the most suitable hyperparameters. The selected hyperparameters for the pretraining process were as follows: initial learning rate: 3e^–5^; warmup learning rate: 1e^–6^; warmup steps: 3000; and optimizer: AdamW.

#### Fine-Tuning Details

The fine-tuning phase involved an extensive process encompassing various BLIP checkpoints and data sets, which included the BLIP original base model checkpoint, BioMedBLIP-ROCO-10, BioMedBLIP-ROCO-20, BioMedBLIP-ROCO-50, BioMedBLIP-MIMIC-10, BioMedBLIP-MIMIC-20, BioMedBLIP-MIMIC-50, BioMedBLIP-ROCO&MIMIC-10, BioMedBLIP-ROCO&MIMIC-20, and BioMedBLIP-ROCO&MIMIC-50. These checkpoints were fine-tuned on a selection of data sets, namely, ImageCLEF, SLAKE, VQA-RAD, PathVQA, Open-I (Indiana University X-RAY), and PEIR-Gross.

For the fine-tuning process, we adhered to the common practice of splitting the data sets into training, validation, and test sets. Typically, the training set constitutes 80% of the total data set, while the test set encompasses the remaining 20%. In certain cases, the data set authors had already performed the necessary data set splits, and we made no further modifications to these sets. In addition, we created an answer list to facilitate the evaluation of predicted VQA generation sentences, VQA classification labels, and image captions.

As part of our methodology, we used a YAML configuration file, which proved essential for adapting to different running environments. Through a series of meticulously designed experiments, we optimized the hyperparameters for each checkpoint’s fine-tuning process. These hyperparameters included train batch size, test batch size, learning rates, and the number of epochs. Importantly, the optimal hyperparameters varied for each checkpoint when applied to different data sets, ensuring the fine-tuning process was meticulously tailored for each specific scenario.

### Resources

This study relied on a combination of hardware and software resources to execute efficiently. We used 3 primary platforms for code execution: Google Colab (Google Inc), Google Cloud Platform (Google Inc), and the University of Sydney’s Artemis HPC supercomputer. Google Colab we used had an Intel(R) Xeon(R) central processing unit (CPU) running at 2.30 GHz, an Nvidia Tesla P100-PCIE-16GB (Nvidia Corp) graphics processing unit (GPU), and 12.8 GB of RAM. The version of the Google Cloud platform we used had 4 CPU cores, a Tesla A100-PCIE-40GB GPU, and 26 GB of RAM. On the Artemis HPC platform [34], we had access to an impressive array of resources, including 7636 CPU cores, 45 TB of RAM, 108 NVIDIA V100 GPUs, 378 TB of storage, and 56 Gbps FDR InfiniBand networking. For our pretraining tasks, we specifically used 4 CPU cores, Tesla V100-PCIE GPUs, and 48 GB of RAM. Our code is available on HuggingFace [7].

### Ethical Considerations

In our study, ethical considerations were meticulously observed to ensure compliance with relevant regulations and standards. We did not require ethics approval for this research because the data sets used, including ROCO, MIMIC-CXR, SLAKE, PathVQA, VQA-RAD, ImageCLEF, Open-I, and PEIR-Gross, are publicly available. These data sets have been previously collected, anonymized, and made accessible for research purposes by their respective organizations and custodians. No user data or human data were collected directly by us for this study. We strictly adhered to data use policies specified by the data providers, ensuring that all data handling was performed in accordance with ethical guidelines. In addition, the data sets used did not contain personally identifiable information, and appropriate measures were taken to maintain data privacy and security throughout the research process. Using publicly available data, we ensured that our research complied with institutional and local policies regarding the use of medical data for research purposes.

## Results

### Overview

In this section, we describe our evaluation metrics along with the results and findings. The purpose of our experimentation was to evaluate how adding domain-specific information helps in improving the performance of downstream tasks in the biomedical domain. For this reason, we have compared the results of our models with the original BLIP model.

### Evaluation Metrics

In this subsection, we provide a detailed overview of the specific evaluation metrics used to assess the performance of our model across various downstream tasks.

- VQA generation: for VQA generation, we used exact match (EM) as the metric. EM assesses the model’s performance by treating predictions that precisely match the ground truth as correct answers. It is particularly relevant for evaluating generative tasks.
- VQA classification metrics: for VQA classification, we used accuracy. Accuracy is a fundamental metric for classification tasks, quantifying the proportion of correctly classified instances.
- Report generation or image captioning metrics: Bilingual Evaluation Understudy (BLEU) is a metric that assesses the similarity between the generated answer and the reference answer, considering n-grams. BLEU-1, in particular, focuses on 1-grams.

### VQA Generation Results

#### Overview

For the VQA eneration task, we used the SLAKE, VQA-RAD, PathVQA, and ImageCLEF data sets to check the performance of our models BLIP-Original, BioMedBLIP-ROCO, and BioMedBLIP-MIMIC&ROCO. These models were designed to generate answers for visual questions and underwent different pretraining strategies. Tables 1 and 2 present a comparison of BioMedBLIP models with the SOTA model.

**Table 1:** Comparison of BioMedBLIP models versus the SOTA^a^ on VQA^b^ generation tasks (part 1).

Data set	Original BLIP^c^ (SOTA; accuracy)	BioMedBLIP models (accuracy)			
		ROCO^d^-10	ROCO-20	ROCO-30	MIMIC^e^-10
SLAKE^f^-Overall	77.95	80.87	80.11	80.21	78.51
SLAKE-Open	73.80	75.81	74.57	75.04	73.80
SLAKE-Close	87.26	88.70	88.70	89.42	85.82
VQA-RAD^g^-Overall	34.37	35.70	*37.03* ^h^	35.03	26.16
VQA-RAD-Open	39.66	43.02	*46.37*	43.02	26.82
VQA-RAD-Close	30.88	30.51	30.88	29.78	25.74
PathVQA-Overall	*66.64*	63.00	64.65	55.46	51.45
PathVQA-Open	*43.78*	38.45	40.79	23.84	18.26
PathVQA-Close	88.35	87.62	*88.57*	87.16	84.75
ImageCLEF^i^	48.20	*58.27*	56.81	57.63	56.41

^a^SOTA: state of the art.

^b^VQA: visual question answering.

^c^BLIP: Bootstrapping Language-Image Pretraining.

^d^ROCO: Radiology Objects in Context.

^e^MIMIC: Medical Information Mart for Intensive Care.

^f^SLAKE: Semantically-Labeled Knowledge-Enhanced.

^g^VQA-RAD: Visual Question Answering in Radiology.

^h^Best performing models are italicized.

^i^ImageCLEF: Image Cross-Language Evaluation Forum.

**Table 2:** Comparison of BioMedBLIP models versus the SOTA^a^ on VQA^b^ generation tasks (part 2).

Data set	BioMedBLIP models (accuracy)				
	MIMIC^c^-20	MIMIC-50	MIMIC&ROCO-10	MIMIC&ROCO-20	MIMIC&ROCO-50
SLAKE^d^-Overall	79.92	70.50	*82.00* ^e^	81.53	81.34
SLAKE-Open	75.50	65.12	76.28	76.28	*76.59*
SLAKE-Close	86.78	78.85	*90.87*	76.28	88.70
VQA-RAD^f^-Overall	30.38	25.50	32.59	29.93	35.70
VQA-RAD-Open	36.87	22.35	32.96	29.05	40.78
VQA-RAD-Close	26.10	27.57	*32.35*	30.51	*32.35*
PathVQA-Overall	53.31	50.89	61.74	54.09	60.27
PathVQA-Open	20.79	17.71	36.22	22.62	33.69
PathVQA-Close	85.91	84.14	87.32	85.64	86.92
ImageCLEF^g^	52.27	53.63	19.89	54.80	56.42

^a^SOTA: state of the art.

^b^VQA: visual question answering.

^c^MIMIC: Medical Information Mart for Intensive Care.

^d^SLAKE: Semantically-Labeled Knowledge-Enhanced.

^e^Best performing models are italicized.

^f^VQA-RAD: Visual Question Answering in Radiology.

^g^ImageCLEF: Image Cross-Language Evaluation Forum.

#### Results on the SLAKE Data Set

For the overall SLAKE data set, the BioMedBLIP MIMIC&ROCO-10 model exhibited the highest EM accuracy, reaching an impressive 82%, outperforming the BLIP original model and other variants. The results in SLAKE-Open highlighted the superiority of the BioMedBLIP MIMIC&ROCO-50 model, which achieved the best performance with an EM accuracy of 76.59%. Finally, in the SLAKE-Close category, the BioMedBLIP MIMIC&ROCO-10 model stood out with an impressive accuracy of 90.87%, demonstrating its strong performance in generating answers that match ground truth answers exactly.

#### Results on the VQA-RAD Data Set

The original BLIP (SOTA) model, serving as the baseline, achieved an EM score of 34.37 in the “VQA-RAD-Overall” category. However, it was surpassed by our BioMedBLIP model, specifically “ROCO-20,” which demonstrated exceptional performance with an EM score of 37.03, indicating its effectiveness in generating accurate answers to visual questions. This trend continued in the “VQA-RAD-Open” data set, where BioMedBLIP-ROCO-20 outperformed the baseline with an EM score of 46.37, highlighting its strong performance in open-ended VQA tasks. Notably, the close-ended category also saw success for the BioMedBLIP models, with “MIMIC&ROCO-10” and “MIMIC&ROCO-50” achieving the top EM score of 32.35.

#### Results on the PathVQA Data Set

In the evaluation of VQA generation tasks on the PathVQA data set, the original BLIP (SOTA) model exhibited an EM score of 66.64, setting a high standard. Among the BioMedBLIP models, “ROCO-20” emerged as the top performer in the PathVQA-Overall and PathVQA-Open categories, achieving scores of 64.65 and 40.79, respectively. Particularly noteworthy is “MIMIC&ROCO-10,” which achieved a competitive EM score of 61.74 in the PathVQA-Overall task. In the PathVQA-Close category, “BioMedBLIP-ROCO-20” stood out with an EM score of 88.57, surpassing the original BLIP (SOTA) model. The BioMedBLIP models “MIMIC&ROCO-10” and “MIMIC&ROCO-50” also displayed a strong performance. These results emphasize the effectiveness of different pretraining strategies and the potential for improved performance in VQA generation tasks using the PathVQA data set with BioMedBLIP.

#### Results on the ImageCLEF Data Set

The SOTA original BLIP model achieved an EM score of 48.20. In contrast, the BioMedBLIP models, which were pretrained with different data sets and epochs, demonstrated notable improvements. Notably, the BioMedBLIP model pretrained with ROCO (10 epochs) emerged as the top performer with an impressive EM score of 58.27, surpassing the SOTA model. This suggests that the use of the ROCO data set for pretraining significantly enhances the ability of the model to generate precise answers to visual questions on the ImageCLEF data set. Other variants of the BioMedBLIP model, pretrained with different data sets and epochs, also exhibited varying degrees of success in this task.

### VQA Classification Results

#### Overview

For the VQA classification task, we used the SLAKE, VQA-RAD, and PathVQA data sets to check the performance of our models BLIP-Original, BioMedBLIP-ROCO, and BioMedBLIP-MIMIC-CXR. These models were designed to generate answers for visual questions and underwent different pretraining strategies. Tables 3 and 4 present a comparison of the BioMedBLIP models with the SOTA models.

**Table 3:** Comparison of BioMedBLIP models versus the SOTA^a^ on VQA^b^ classification tasks (part 1).

Data set	Original BLIP^c^ (SOTA; accuracy)	BioMedBLIP models (accuracy)			
		ROCO^d^-10	ROCO-20	ROCO-30	MIMIC^e^-10
SLAKE^f^-Overall	77.85	*81.06* ^g^	80.21	80.04	78.70
SLAKE-Open	75.50	75.66	77.05	77.52	75.66
SLAKE-Close	81.49	83.31	*85.10*	84.86	83.41
VQA-RAD^h^-Overall	*40.35*	33.70	19.96	23.95	34.36
VQA-RAD-Open	20.67	27.37	25.10	26.33	*28.49*
VQA-RAD-Close	*51.84*	39.71	33.09	39.71	38.23
PathVQA-Overall	60.09	59.25	57.77	58.65	58.85
PathVQA-Open	*37.21*	33.60	30.06	33.17	34.05
PathVQA-Close	85.15	84.96	85.54	84.20	83.71

^a^SOTA: state of the art.

^b^VQA: visual question answering.

^c^BLIP: Bootstrapping Language-Image Pretraining.

^d^ROCO: Radiology Objects in Context.

^e^MIMIC: Medical Information Mart for Intensive Care.

^f^SLAKE: Semantically-Labeled Knowledge-Enhanced.

^g^Best performing models are italicized.

^h^VQA-RAD: Visual Question Answering in Radiology.

**Table 4:** Comparison of BioMedBLIP models versus the SOTA^a^ on VQA^b^ classification tasks (part 2).

Data set	BioMedBLIP models (accuracy)				
	MIMIC^c^-20	MIMIC-50	MIMIC&ROCO^d^-10	MIMIC&ROCO-20	MIMIC&ROCO-50
SLAKE^e^-Overall	77.57	74.18	73.90	80.89	69.10
SLAKE-Open	74.88	72.25	71.62	*77.90* ^f^	71.32
SLAKE-Close	81.73	77.16	76.69	84.77	68.04
VQA-RAD^g^-Overall	33.70	34.15	34.59	29.49	31.49
VQA-RAD-Open	*28.49*	*28.49*	*28.49*	26.26	27.93
VQA-RAD-Close	37.13	37.87	38.69	31.62	33.82
PathVQA-Overall	58.04	36.86	60.41	60.24	*61.13*
PathVQA-Open	31.98	18.96	32.61	33.32	34.09
PathVQA-Close	84.17	54.80	59.70	85.38	*86.29*

^a^SOTA: state of the art.

^b^VQA: visual question answering.

^c^MIMIC: Medical Information Mart for Intensive Care.

^d^ROCO: Radiology Objects in Context.

^e^SLAKE: Semantically-Labeled Knowledge-Enhanced.

^f^Best performing models are italicized.

^g^VQA-RAD: Visual Question Answering in Radiology.

#### Results on the SLAKE Data Set

In the context of VQA classification tasks using the SLAKE data set, the evaluation results are presented in terms of accuracy, allowing for a comprehensive comparison between the original BLIP model and several BioMedBLIP variants, each pretrained with specific data sets and epochs. The BioMedBLIP models demonstrated their potential for significant improvements over the original BLIP model. In the “SLAKE-Overall” data set category, BioMedBLIP models pretrained with the ROCO data set consistently outperformed the original BLIP model, with ROCO-10 achieving an accuracy of 81.06. Furthermore, BioMedBLIP models pretrained with the MIMIC-CXR data set, particularly MIMIC-20, showcased strong performance. For the SLAKE open-ended data set subtask, the model pretrained with both MIMIC-CXR and ROCO for 20 epochs achieved an accuracy of 77.90, surpassing the original BLIP model. For the SLAKE close-ended data set, the ROCO-20 variant of BioMedBLIP stood out with an accuracy of 85.10, clearly surpassing the original BLIP model’s performance. These results emphasize the significance of data set choice and pretraining duration, with BioMedBLIP models showcasing their potential for improved accuracy in classifying visual questions within the SLAKE data set.

#### Results on the VQA-RAD Data Set

In the VQA-RAD-Overall data set, encompassing both open-ended and closed-ended questions, none of the BioMedBLIP models outperformed the original BLIP model, highlighting the challenges in achieving superior accuracy in a mixed question type. However, in the VQA-RAD open-ended data set, all the BioMedBLIP models excelled, surpassing the original BLIP model’s accuracy and showcasing their effectiveness in open-ended question answering. Four variants of BioMedBLIP models, namely, MIMIC-10, MIMIC-20, MIMIC-50, and MIMIC&ROCO-10, had the highest score of 28.49. For the VQA-RAD close-ended data set, the BioMedBLIP models did not surpass the original BLIP model, with the best performers being ROCO-10 and ROCO-30. This shows that further research is warranted on strategies to improve the performance on the close-ended VQA-RAD data set.

#### Results on the PathVQA Data Set

On the PathVQA-Overall data set, the original BLIP model achieved an accuracy of 60.09, while the BioMedBLIP models demonstrated varied performance. Notably, the model pretrained with both MIMIC-CXR and ROCO data sets for 50 epochs emerged as the top performer, achieving an accuracy of 61.13. This dual pretraining approach showed significant promise in enhancing classification accuracy. For the PathVQA-Open data set, the original BLIP model achieved the highest accuracy of 37.21, with BioMedBLIP models pretrained on ROCO and MIMIC-CXR data yielding slightly lower results. The highest performance among BioMedBLIP models came from the model pretrained with both data sets for 50 epochs, reaching an accuracy of 34.09, which is very close to the original BLIP model’s accuracy. In contrast, the original BLIP model excelled on the PathVQA-Close data set, achieving an impressive accuracy of 85.15. Nevertheless, the BioMedBLIP model pretrained with both MIMIC-CXR and ROCO for 50 epochs outperformed the original BLIP model, achieving an accuracy of 86.29.

These results collectively indicate that the choice of pretraining strategy and the duration of training significantly influence the classification accuracy of BioMedBLIP models on the various VQA classification data sets, with the combined data set pretraining demonstrating notable advantages in certain contexts.

### Image Captioning Task Results

We used the PEIR-Gross data set for the image captioning task to check the performance of our BioMedBLIP models. For the image captioning task, BLIP-original had a BLEU-1 score of 24.8. Similarly, when using the ROCO data set for training, the BioMedBLIP models had scores of 24.4, 24.6, and 25.1 for 10, 20, and 30 epochs respectively. Furthermore, when the MIMIC data set was used, the BLEU-1 score of 23.1, 23.9, and 24.1 was achieved by BioMedBLIP models when trained for 10, 20, and 50 epochs respectively. Additionally, when the training data combined MIMIC and ROCO, the BLEU-1 score of 23.9, 24.3, and 24.2 was achieved for 10, 20, and 30 epochs respectively by BioMedBLIP models. In terms of BLEU-1 scores, higher values are indicative of better performance. The results show that the BioMedBLIP-ROCO-50 model surpassed the original BLIP model in the image captioning task with a BLEU-1 score of 25.1 (over 1.2% improvement from the score of the original BLIP model), demonstrating that our approach has the potential to enhance the model’s capabilities for generating captions. In contrast, all other models, including various pretraining strategies and epochs, exhibited slightly lower BLEU-1 scores. While some models may have exhibited minor variations in performance, it is essential to emphasize that, overall, our results were quite consistent. This consistency indicates that our pretraining strategies and fine-tuning approaches are robust and capable of producing reliable outcomes.

## Discussion

### Principal Findings

In the presented VQA generation, VQA classification, and report generation tasks, we aimed to assess the performance of our BioMedBLIP models against the SOTA original BLIP model using diverse medical image data sets and pretraining strategies. The findings indicate that our BioMedBLIP models, pretrained with specialized medical data sets, exhibit substantial improvements in generating answers for visual questions, as well as in classifying images and questions, depending on the specific data set and pretraining strategy used.

In this section, it is important to emphasize the general trends and insights that can be drawn from these results:

- Data set specificity: the choice of pretraining data sets, such as ROCO and MIMIC-CXR, significantly impacts model performance in both VQA generation and VQA classification tasks. Specialized medical data sets have proven to be valuable for enhancing model capabilities in medical image analysis and question answering.
- Pretraining duration: longer pretraining durations, as evidenced by models such as MIMIC&ROCO-50, have shown their potential to improve classification accuracy in specific categories of questions, demonstrating the importance of considering pretraining strategies tailored to the task.
- Diverse performance: our models exhibit varying levels of success across different data sets and task categories. This underscores the need for flexibility in selecting pretraining strategies, depending on the specific goals and data sets of a given application.
- Image captioning: our approach, particularly the one followed for BioMedBLIP-ROCO-50, demonstrates improvements in generating captions for medical images, showcasing the model’s capacity to excel in both VQA and image captioning tasks.
- Selection of epoch numbers: in our experiments, we observed that the BioMedBLIP models converge at varying numbers of epochs, depending on the data set and the specific downstream tasks involved. This variability is typical in deep learning, where a model’s loss decreases up to a certain point and then may increase, indicating that longer training periods do not necessarily yield better performance.

These results underscore the potential of our BioMedBLIP models to excel in a wide range of medical image analysis and question answering tasks, with their performance varying depending on the specific data set and task at hand. BioMedBLIP was tested using 20 different data sets and task combinations. Our method excelled in 15 (75%) out of 20 tasks. BioMedBLIP represents a new SOTA in 15 (75%) out of 20 tasks, and our responses were rated higher in all 20 tasks (*P*<.005) in comparison to SOTA models. Regression analyses showed that our model’s VQA generation has a statistically significant predictor (*P*<.002) on the SLAKE, PathVQA, and ImageCLEF data sets. In contrast, our model’s VQA classification has a relatively lower predictor (*P*<.003) on the SLAKE, PathVQA, and VQA-RAD data sets in accordance with the regression analyses.

### VQA Generation

For the SLAKE data set, our model, pretrained on a combination of general domain data sets and medical domain data sets (MIMIC-CXR and ROCO), consistently outperformed other models across various SLAKE data sets, including open-ended, close-ended, and aggregated types. In contrast to the study by Li et al [12], our results affirm the benefits of pretraining on both general and medical data sets, addressing limitations in their work. This underscores the advantage of a domain-specific model for specialized downstream tasks. Notably, our observations align with the findings of Eslami et al [35], indicating that a pretrained ViT, such as our BLIP model, possesses a comprehensive understanding of image content and long-range dependencies, essential for interpreting the SLAKE data set. Surprisingly, in the VQA-RAD data set, the model pretrained solely on the ROCO data set (for 20 epochs) excelled in open-ended and aggregated tasks, while the MIMIC-CXR and ROCO model performed better in the close-ended task. Contrary to expectations, the model pretrained on general domain data sets and ROCO outperformed the model pretrained on larger domain-specific data sets for PathVQA and ImageCLEF. We attribute this to the superior preprocessing of the ROCO data set, incorporating red bounding boxes that aid in learning crucial image regions. Moreover, our 50-epoch pretraining might not suffice for larger data sets, suggesting the need for further exploration with extended training. Overall, our models demonstrated superior performance on the medical data sets compared to the original BLIP model, emphasizing the efficacy of our approach in medical image analysis.

### VQA Classification

In the exploration of VQA classification tasks, diverse models underwent experimentation and fine-tuning across data sets such as SLAKE, PathVQA, and VQA-RAD, encompassing open-ended, close-ended, and aggregated question types. Notably, the SLAKE data set consistently emerged as the data set where BioMedBLIP models consistently exhibited superior performance. Tables 3 and 4 highlight that, in the majority of cases, the BioMedBLIP models performed better than the original BLIP model.

Furthermore, our experiments delved into the impact of different epoch settings on model performance. An intriguing observation emerged, indicating that the relationship between a model’s performance and the number of epochs is not consistently positive. This suggests the critical importance of judiciously selecting epoch configurations during the construction of visual language models for medical data sets, challenging the notion that more epochs always lead to improved accuracy.

### Image Captioning

In the context of the image captioning task, our findings, while not entirely satisfactory, reveal promising aspects, particularly with the BLIP-ROCO 50 model. This variant surpasses the original BLIP model in BLEU measurements, hinting at potential improvements in using the BLIP model for image captioning. However, the overall performance of the modified models hovers around 23% to 25%, suggesting that the BLIP model may not be inherently well suited for image captioning tasks.

### Further Insights

The development and application of our BioMedBLIP models have far-reaching implications across the health care and educational sectors. First and foremost, our models can significantly contribute to improving medical diagnosis and decision support systems. By enhancing the capacity to analyze medical images and answer visual questions, they have the potential to facilitate more accurate and timely health care interventions, ultimately benefiting patient outcomes. In medical education and training, our models can serve as valuable tools for students and professionals alike. Automated question answering capabilities can bridge knowledge gaps and improve learning outcomes in a field that demands continuous learning. Moreover, the automation of image analysis and question answering tasks has the potential to reduce the workload on medical professionals, allowing them to allocate more time to complex aspects of patient care. In terms of research, our models can expedite medical investigations by streamlining the analysis of extensive data sets, potentially leading to groundbreaking discoveries and advancements in the field. Finally, on a global scale, the availability of advanced artificial intelligence models such as ours can improve medical services in underserved regions where access to specialized medical expertise is limited, thereby contributing to more equitable health care delivery. However, these opportunities are accompanied by ethical and societal responsibilities. Ensuring patient privacy, addressing biases in the data, and maintaining transparency in the development and deployment of artificial intelligence models are pivotal steps to maximize their positive impact while mitigating potential risks and pitfalls in the medical field and beyond.

Our BioMedBLIP models, while showing promise in medical image analysis, come with inherent limitations. First, their performance is heavily contingent on the quality and diversity of the training data. Limitations in data availability, such as smaller or less representative medical data sets, can hinder the model’s ability to generalize to real-world scenarios and may introduce biases. Second, the variability in model performance across different data sets and task categories poses a challenge. Achieving optimal results often demands the fine-tuning of pretraining strategies for specific tasks, which may not always be straightforward in practical applications. While choosing the models appropriate for real-world applications, there are various considerations to be made. In our work, we have presented widely used metrics to evaluate model’s performance. Different downstream tasks might have different metrics that are used for evaluation. In this case, comparison on the grounds of the most relevant metrics should be made. Moreover, the computational demands for pretraining models, especially in the context of medical tasks, can be substantial, potentially limiting the accessibility of our approach to settings with limited computational resources. We recommend training models for different epochs before selecting them for real-world applications. This is because for different data sets and tasks, the models tend to show convergence at different numbers of epochs. Finally, ethical and privacy concerns are paramount in the use of medical image data. Striving to ensure strict compliance with data protection regulations and maintaining patient privacy and data security are imperative in any real-world implementation.

### Conclusions

In conclusion, our development and evaluation of BioMedBLIP models for medical image analysis tasks reveal both promise and practical considerations. These models have shown substantial potential in enhancing the interpretation of medical images and responding to visual questions in a health care context. The choice of pretraining data sets, including ROCO and MIMIC-CXR, plays a pivotal role in model performance, underscoring the importance of specialized medical data for training. Furthermore, a longer duration of pretraining, exemplified by the MIMIC&ROCO-50 model, has demonstrated the potential to elevate classification accuracy in specific question categories. However, our findings highlight the variability in performance across different data sets and tasks, necessitating a flexible approach to pretraining strategies. Moreover, our models have promising implications across health care and education. They can bolster medical diagnosis, decision support systems, and research efforts while also streamlining medical education and reducing the workload on health care professionals. The global accessibility of these models can bring specialized medical expertise to underserved regions.

## Abbreviations

BERT: Bidirectional Encoder Representations From Transformers
BLEU: Bilingual Evaluation Understudy
BLIP: Bootstrapping Language-Image Pretraining
CLIP: Contrastive Language-Image Pretraining
CPU: central processing unit
CT: computed tomography
EM: exact match
GPU: graphics processing unit
ImageCLEF: Image Cross-Language Evaluation Forum
LXMERT: Learning Cross-Modality Encoder Representations From Transformers
MedVQA: medical visual question answering
MIMIC-CXR: Medical Information Mart for Intensive Care-Chest X-ray
MRI: magnetic resonance imaging
NLP: natural language processing
PEIR: Pathology Education Informational Resource
ROCO: Radiology Objects in Context
SLAKE: Semantically-Labeled Knowledge-Enhanced
SOTA: state of the art
V+L: vision and language
ViT: Vision Transformer
VQA: visual question answering
